# Lactate/albumin ratio as a predictor of in-hospital mortality in critically ill children

**DOI:** 10.1186/s12887-022-03787-0

**Published:** 2022-12-20

**Authors:** Guan Wang, Junhui Liu, Rui Xu, Yanan Fu, Xinjie Liu

**Affiliations:** 1 Department of Pediatrics, Qilu Hospital of Shandong University, No.107 West Wenhua Road, 250012 Jinan, Shandong Province China; 2 Department of Medical Engineering, Qilu Hospital of Shandong University, No.107 West Wenhua Road, 250012 Jinan, Shandong Province China

**Keywords:** Critically ill children, Lactate/albumin ratio, Lactate, Albumin, PICU, In-hospital mortality

## Abstract

**Objective:**

Managing critically ill patients with high mortality can be difficult for clinicians in pediatric intensive care units (PICU), which need to identify appropriate predictive biomarkers. The lactate/albumin (L/A) ratio can precisely stratify critically ill adults. However, the role of the L/A ratio in predicting the outcomes of critically ill children remains unclear. Therefore, this study aimed to evaluate the prognostic performance of the L/A ratio in predicting in-hospital mortality in unselected critically ill patients in the PICU.

**Methods:**

This was a single-center retrospective study. Clinical data of 8,832 critical patients aged between 28 days and 18 years were collected from the pediatric intensive care (PIC) database from 2010 to 2018. The primary outcome was the in-hospital mortality rate.

**Results:**

There was a higher level of L/A ratio in non-survivors than survivors (*P* < 0.001). Logistic regression indicated that the association between the L/A ratio and in-hospital mortality was statistically significant (OR 1.44, 95% CI 1.31–1.59, *P* < 0.001). The AUROC of the L/A ratio for predicting in-hospital mortality was higher than lactate level alone (0.74 vs 0.70, *P* < 0.001). Stratification analysis showed a significant association between the L/A ratio and in-hospital mortality in the age and primary disease groups (*P* < 0.05).

**Conclusions:**

Our study suggested that the L/A ratio was a clinical tool to predict in-hospital mortality in critically ill children better than lactate level alone. However, given that the study was retrospective, more prospective studies should be conducted to test the predictive value of the L/A ratio in critical illness.

**Supplementary Information:**

The online version contains supplementary material available at 10.1186/s12887-022-03787-0.

## Introduction

Patients with high mortality in pediatric intensive care units (PICU) occupy the majority of resources. Despite China’s progress in intensive care and critical illness management, the overall mortality rate in PICU is much higher than in developed countries [1]. Therefore, it is crucial to identify predictive death biomarkers in critically ill children for early detection and timely treatment.

Because of tissue hypoxia and anaerobic metabolism, elevated serum lactate levels reflect poor tissue perfusion and illness severity [2]. Recent studies have shown that increased lactate levels are associated with poor outcomes and elevated mortality in patients with sepsis [3–5]. However, lactate levels are also affected by other factors such as lactate clearance from the liver and aerobic glycolysis via Na-K ATPase [6]. Patients with diabetic ketoacidosis, renal dysfunction, metabolic disorders, tumors, and intoxication also develop lactic acidosis of varying degrees [7–10]. Non-pathogenic factors, such as some medications like epinephrine, metformin, and linezolid also can lead to elevated lactate levels. In addition, some patients with normal or moderate lactate levels are at high risk of death [11–14]. In these cases, it is challenging and sometimes unreasonable relying solely on lactate levels to predict the prognosis of critical illness.

The lactate/albumin (L/A) ratio is an emerging prognostic biomarker that combines lactate with albumin levels. As a principal negative acute-phase protein, albumin is mainly synthesized by the liver and serves several vital physiological functions [15]. It is affected by nutritional status, inflammation, and chronic diseases [16, 17], especially liver dysfunction. The L/A ratio may provide a variable consisting of comprehensive information regarding nutrition and other physiological changes in patients. Some studies have demonstrated that the L/A ratio is associated with multiple organ failure and mortality in patients with severe sepsis or septic shock [18, 19]. Studies on the L/A ratio for predicting the prognosis of critical illnesses have received increasing attention, and some studies based on small sample data have been reported [2, 20], mainly focusing on adults. However, the role of the L/A ratio in predicting outcomes in critically ill children remains unclear, and further validation is required before clinical practice. The present study aimed to investigate the prognostic performance of the L/A ratio in predicting in-hospital mortality in unselected critically ill patients admitted to the PICU.

## Methods

### Study design

We collected 8,832 patients’ clinical data from the pediatric intensive care (PIC) database (version 1.1.0), a large China-based pediatric critical care database [21]. As an integrated, de-identified, and comprehensive clinical dataset, the PIC contains hospital clinical records from 2010 to 2018 at the Children’s Hospital, Zhejiang University School of Medicine. The primary cohort included 12,881 patients with 13,941 ICU admissions. The survey protocol was approved by the Institutional Review Board of the Children’s Hospital, Zhejiang University School of Medicine (Hangzhou, China). The requirement for individual patient consent was waived because the study did not impact clinical care, and all protected health information was de-identified.

### Study participants

**Fig. 1:**
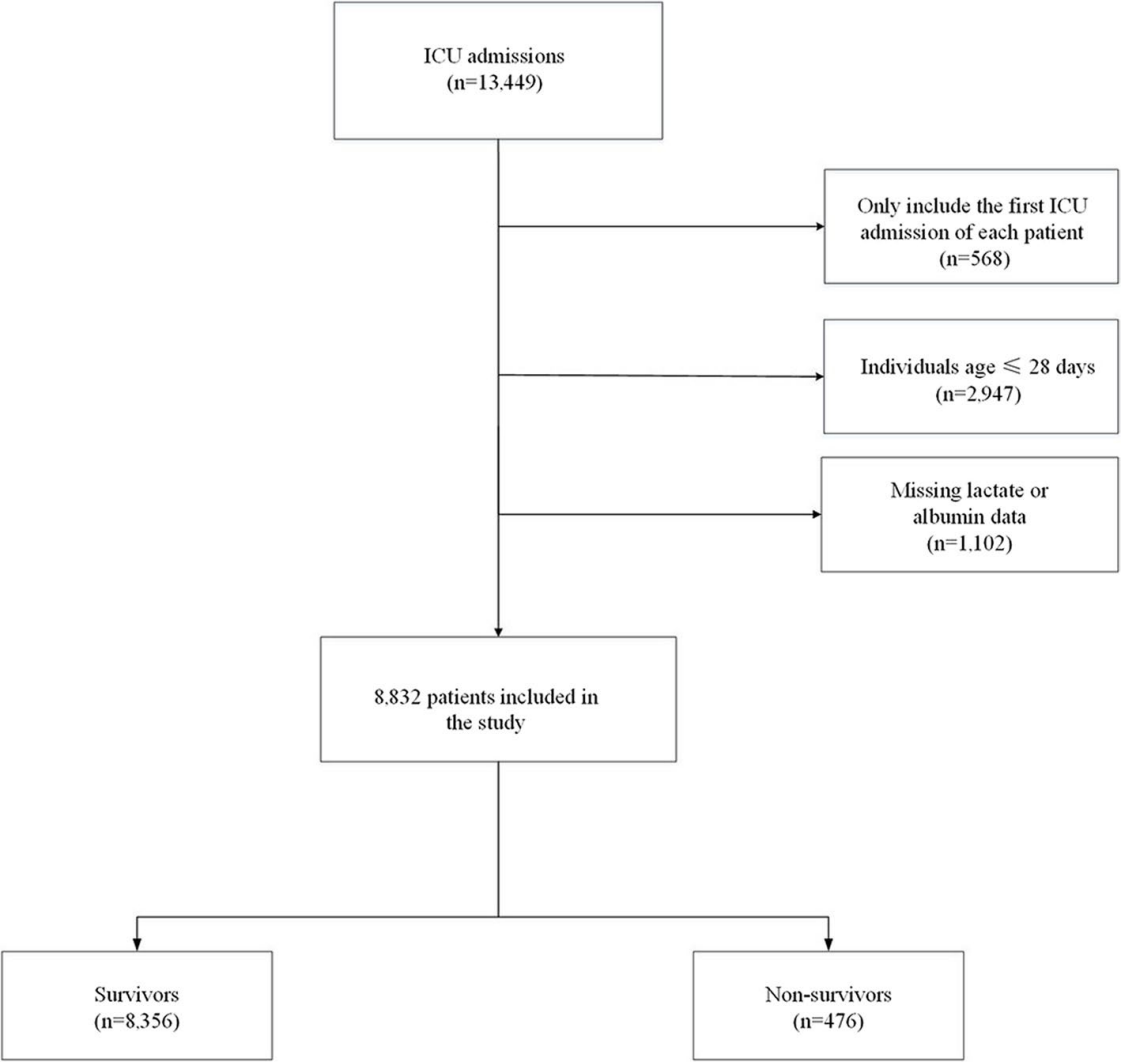
Flow chart of recruitment of the study population

Individuals aged ≤ 28 days or > 18 years, and individuals with missing lactate or albumin data were not included in this study. If an individual had two or more PICU admissions, we only collected clinical data on the first PICU admission. The final cohort contained 8,832 patients. Among them, 8,356 patients who survived until discharge from the hospital were defined as survivors, and 476 patients who died in the hospital were included in the non-survivor group (Fig. 1).

### Data collection

The clinical data collected included patient characteristics, vital signs, laboratory results, treatments (vasopressor use, such as dopamine and epinephrine), and mortality outcomes. All laboratory variables were obtained from the first blood withdrawn after PICU admission. Hospital diagnoses were assigned using the International Statistical Classification of Diseases and Related Health Problems, 10th (ICD-10) system. The primary outcome was in-hospital mortality.

### Statistical analysis

Shapiro-Wilk test was used to test the normal distributions of variables. Continuous variables with normal distribution were tested by Student’s *t* test and presented as the mean ± standard deviation (SD), whereas non-normal distributed continuous variables were tested by Mann–Whitney U-test and presented as the median with interquartile range (IQR, Q1-Q3). Categorical variables were tested by chi-square analysis or Fisher’s exact test and described as number (percentage). Multivariable logistic regression models were built to adjust for potential confounders in the association between the L/A ratio and in-hospital mortality (primary outcome), which were shown as odds ratios (ORs) with 95% confidence intervals (CIs). Model I was unadjusted. In Model II, all variables with statistical and clinical significance were included in the analysis, including age, sex, ICU type, bacteremia, vasopressor use, white blood cell (WBC), platelet (PLT), hemoglobin, alanine transaminase (ALT), creatine kinase (CK)-MB, sodium, international normalized ratio (INR), and C-reactive protein (CRP). Receiver operating characteristic (ROC) analysis was performed, and the area under the curve (AUC) was calculated to evaluate the predictive value of the L/A ratio in critical illness in children. MedCalc software version 14.8.1 (MedCalc Software Ltd, Ostend, Belgium) was used to test the statistical difference in AUCs by DeLong method. Youden’s index was calculated to determine the optimal cut-off values that predict in-hospital mortality. Subgroup analyses were also performed to assess the AUC of both lactate and the L/A ratio in patients with different ages and lactate levels, bacteremia, and different primary diagnoses. A stratification analysis was conducted to examine whether the association between the L/A ratio and in-hospital mortality differed across various subgroups classified by age, sex, bacteremia, lactate levels, and primary diagnosis. Statistical analyses were conducted using R version 3.4.3 (https://www.r-project.org, The R Foundation for Statistical Computing, Vienna, Austria) and EmpowerStats (http://www.empowerstats.com, X&Y Solutions, Inc., Boston, Mass, USA) software packages. Statistical significance was defined as a two-sided *P*-value of < 0.05.

## Results

### Clinical characteristics of the study population

The clinical characteristics of survivors and non-survivors are shown in Table 1. There were no significant differences in age between the two groups. However, compared with the survivor group, there was a higher proportion of boys in the non-survivor group (62.4%, *P* = 0.004). Compared to the survivor group, the non–survivor group had higher heart and respiratory rates as well as lower systolic blood pressure (SBP) and oxygen saturation (SpO2) (all *P* < 0.05). However, there were no significant differences in other vital signs, including temperature and diastolic blood pressure (both *P* > 0.05). The non-survivors included in this study mainly came from the General intensive care unit (GICU) (45.4%) and the surgical intensive care unit (SICU) (28.1%) provided the most survivors. The non-survivor group had longer ICU days than the survivor group (median 5.5, IQR 1.9–17.3 vs. median 1.8, IQR 0.9–5.5, *P* < 0.001). We also analyzed the type of disease in all participants included in the study. Respiratory diseases were the most common type of illness in the non-survivor group (23.3%). We also found a significant difference in the incidence of bacteremia between the non-survivor and survivor groups (32.5% vs. 18.1%, *P* < 0.001). In addition, there was a higher rate of vasopressor use in the non-survivor group than in the survivor group.

**Table 1:** Clinical characteristics of the study population

Characteristics	Total	Survivors	Non-survivors	*P* value
N	8832	8356	476	
Age (month), median (IQR)	18.0 (5.0–58.0)	18.0 (5.0–59.0)	14.0 (4.0-51.3)	0.263
Gender (male,%)	4952 (56.1%)	4655 (55.7%)	297 (62.4%)	0.004
Vital signs, mean ± SD				
Temperature, ^o^C	36.9 ± 5.4	36.9 ± 5.5	36.7 ± 1.5	0.448
Heart rate (HR, beats/min)	124.6 ± 24.2	124.2 ± 23.9	134.4 ± 30.5	< 0.001
Respiratory rate (RR, breaths/min)	31.4 ± 10.2	31.2 ± 10.1	37.2 ± 13.3	< 0.001
Systolic pressure (SBP, mmHg)	99.5 ± 18.7	99.6 ± 18.6	96.2 ± 19.0	0.014
Diastolic pressure (DBP, mmHg)	57.9 ± 14.2	57.9 ± 14.1	57.2 ± 16.2	0.552
Oxygen saturation (SpO_2_, %)	97.9 ± 4.1	97.9 ± 4.1	95.6 ± 6.3	< 0.001
ICU type, n (%)				< 0.001
CICU	2329 (26.4%)	2304 (27.6%)	25 (5.3%)	
GICU	1871 (21.2%)	1655 (19.8%)	216 (45.4%)	
NICU	413 (4.7%)	393 (4.7%)	20 (4.2%)	
PICU	1825 (20.7%)	1656 (19.8%)	169 (35.5%)	
SICU	2394 (27.1%)	2348 (28.1%)	46 (9.7%)	
Hospital days, median (IQR)	12.0 (7.0-18.9)	12.0 (7.1–18.9)	6.4 (2.2–19.0)	< 0.001
ICU days, median (IQR)	1.9 (0.9–5.9)	1.8 (0.9–5.5)	5.5 (1.9–17.3)	< 0.001
Primary diagnosis on ICU admission, n (%)				< 0.001
Congenital	1249 (14.1%)	1223 (14.6%)	26 (5.5%)	
Hematological	356 (4.0%)	306 (3.7%)	50 (10.5%)	
Circulation	2203 (24.9%)	2132 (25.5%)	71 (14.9%)	
Neurologic	906 (10.3%)	838 (10.0%)	68 (14.3%)	
Digestive	738 (8.4%)	709 (8.5%)	29 (6.1%)	
Neoplasm	801 (9.1%)	775 (9.3%)	26 (5.5%)	
Respiratory	1044 (11.8%)	933 (11.2%)	111 (23.3%)	
Trauma	467 (5.3%)	425 (5.1%)	42 (8.8%)	
Others	1068 (12.1%)	1015 (12.2%)	53 (11.1%)	
Bacteremia, n (%)				< 0.001
No	6878 (81.2%)	6566 (82.0%)	312 (67.5%)	
Yes	1596 (18.8%)	1446 (18.1%)	150 (32.5%)	
Vasopressors, n (%)				< 0.001
No	1704 (19.3%)	1676 (20.1%)	28 (5.9%)	
Yes	3061 (34.7%)	2820 (33.8%)	241 (50.6%)	
Unknown	4067 (46.1%)	3860 (46.2%)	207 (43.5%)	

*ICU* intensive care unit, *CICU* cardiac intensive care unit, *GICU* general intensive care unit, *NICU* neonatal intensive care unit, *PICU* pediatric intensive care unit, *SICU* surgical intensive care unit, *IQR* interquartile range

The laboratory data of survivors and non-survivors are shown in Table 2. Higher levels of WBC, CRP, ALT, CK-MB, INR, and lower levels of hemoglobin, PLT were found in the non-survivor group than survivor group (all *P* < 0.001), which indicated severe inflammation, liver dysfunction, heart insufficiency, and poor coagulation.

**Table 2:** Laboratory information of the study population

Characteristics	N	Total	Survivors	Non-survivors	*P* value
WBC (×10^9^ /L)	8822	9.0 (6.9–12.1)	9.0 (6.9–12.0)	10.2 (6.4–16.2)	< 0.001
PLT (×10^9^ /L)	8822	317.0 (235.0-400.0)	320.0 (241.0-402.0)	229.0 (113.5–351.0)	< 0.001
Hemoglobin (g/L)	8822	116.0 (102.0-126.0)	116.0 (102.0-127.0)	107.0 (89.0-122.0)	< 0.001
ALT (U/L)	8832	20.0 (13.0–35.0)	20.0 (13.0–33.0)	34.0 (18.0-102.3)	< 0.001
CK-MB (U/L)	8805	31.0 (22.0–45.0)	31.0 (22.0–44.0)	35.0 (20.0–75.0)	< 0.001
Creatinine (umol/L)	8830	43.0 (37.0–52.0)	43.0 (37.0–51.0)	47.0 (35.0-65.1)	0.565
Sodium (mmol/L)	8832	136.0 (134.0-139.0)	136.0 (134.0-139.0)	137.0 (133.0-141.0)	0.020
INR	8497	1.0 (0.9–1.1)	1.0 (0.9–1.1)	1.2 (1.0-1.5)	< 0.001
CRP (mg/L)	7350	6.0 (3.0–28.0)	6.0 (3.0–27.0)	11.0 (4.0-44.5)	< 0.001
Lactate (mmol/L)	8832	1.6 (1.1–2.6)	1.6 (1.1–2.5)	2.8 (1.6–5.6)	< 0.001
Albumin (g/dL)	8832	41.8 (37.2–45.2)	42.0 (37.5–45.3)	36.4 (29.9–40.7)	< 0.001
L/A ratio	8832	0.4 (0.3–0.7)	0.4 (0.3–0.6)	0.8 (0.4–1.7)	< 0.001

Values are expressed as median (IQR, Q1-Q3)

*WBC* white blood cell, *PLT* platelet, *ALT* alanine transaminase, *CK-MB* creatine kinase-MB, *INR* international normalized ratio, *CRP* C reactive protein, *L/A ratio* lactate/albumin ratio

Higher lactate levels and lower albumin levels were observed in the non-survivor group than survivor group (Lactate: median 2.8, IQR 1.6–5.6 vs. median 1.6, IQR 1.1–2.5, *P* < 0.001; Albumin: median 36.4, IQR 29.9–40.7 vs. median 42.0, IQR 37.5–45.3, *P* < 0.001). Most importantly, our findings showed a higher L/A ratio in the non-survivor group compared with the survivor group (median 0.8, IQR 0.4–1.7 vs. median 0.4, IQR 0.3–0.6, *P* < 0.001)

### L/A ratio is an independent prognostic factor in critically ill children

We used a multivariable logistic regression model to explore the association between the L/A ratio, lactate, albumin, and in-hospital mortality in critically ill patients. The association between the L/A ratio and hospital mortality was statistically significant in the non-adjusted model (OR 2.02, 95% CI 1.86–2.19, *P* < 0.001). After adjusting for age, sex, ICU type, bacteremia, vasopressor use, WBC, PLT, hemoglobin, ALT, CK-MB, sodium, INR, and CRP, a strong association was observed between the L/A ratio and hospital mortality (OR 1.44, 95% CI 1.31–1.59, *P* < 0.001). This indicated that with increasing L/A ratio, the hospital mortality increases. According to the results, the L/A ratio remained an independent prognostic factor for in-hospital mortality in critically ill children (Table 3). The relationship between the potential predictive variables and in-hospital mortality in different models of multivariable logistic regression were shown in supplementary Table 1.

**Table 3:** Relationship between lactate/albumin (L/A) ratio, lactate, albumin and in-hospital mortality in different models of multivariable logistic regression

	Model I			Model II		
	OR	95% CI	*P*	OR	95% CI	*P*
L/A ratio	2.02	(1.86, 2.19)	< 0.001	1.44	(1.31, 1.59)	< 0.001
Lactate	1.27	(1.23, 1.30)	< 0.001	1.16	(1.12, 1.21)	< 0.001
Albumin	0.90	(0.89, 0.91)	< 0.001	0.95	(0.93, 0.97)	< 0.001

Model I: non-adjusted model

Model II: adjusted for age, sex, ICU type, bacteremia, vasopressors use, WBC, PLT, hemoglobin, ALT, CK-MB, sodium, INR and CRP

*OR* odds ratio, *CI* confidence interval

### Predictive performance of the L/A ratio for in-hospital mortality

Table 4 shows the AUROC of hospital mortality, cut-off value, sensitivity, and specificity of the L/A ratio among the different subgroups. The overall AUROC of the L/A ratio for predicting hospital mortality was higher compared with that of lactate alone (0.74, 95% CI 0.71–0.76 vs. 0.70, 95% CI 0.67–0.73, *P* < 0.001). In addition, the overall cut-off value, sensitivity, and specificity for the L/A ratio were 0.55, 0.66, and 0.69, respectively, which indicated that when the L/A ratio was more than 0.55, there was a chance of developing a poor prognosis in critically ill children.

**Table 4:** Area under the receiver operating characteristic curve (AUROC) and lactate/albumin ratio cut-off for hospital mortality among different patient subgroups

	AUROC curve of hospital mortality (95% confidence interval)			Lactate/albumin ratio cut-off point		
	Lactate	Lactate/albumin ratio	*P*	Cut-off value	Sensitivity	Specificity
Overall	0.70 (0.67, 0.73)	0.74 (0.71, 0.76)	< 0.001	0.55	0.66	0.69
Age						
< 12 months	0.66 (0.62, 0.70)	0.68 (0.65, 0.72)	< 0.001	0.61	0.60	0.65
12 ≤ x < 36 months	0.77 (0.72, 0.81)	0.81 (0.77, 0.85)	< 0.001	0.61	0.67	0.79
36 ≤ x < 120 months	0.71 (0.65, 0.77)	0.74 (0.69, 0.80)	< 0.001	0.54	0.64	0.75
≥ 120 months	0.67 (0.58, 0.76)	0.73 (0.65, 0.81)	0.004	0.55	0.73	0.71
Bacteremia						
No	0.72 (0.69, 0.75)	0.76 (0.73, 0.78)	< 0.001	0.63	0.61	0.77
Yes	0.60 (0.55, 0.65)	0.63 (0.58, 0.67)	0.006	0.61	0.59	0.61
Lactate levels						
Normal lactate (< 2.0 mmol/L)	0.70 (0.67, 0.73)	0.73 (0.70, 0.77)	< 0.001	0.55	0.67	0.68
Intermediate lactate (2.0 ≤ x < 4.0 mmol/L)	0.71 (0.66, 0.76)	0.74 (0.70, 0.79)	< 0.001	0.69	0.57	0.78
High lactate ( ≥ 4.0 mmol/L)	0.68 (0.58, 0.77)	0.73 (0.64, 0.81)	0.004	0.54	0.67	0.70
Primary diagnosis						
Congenital	0.56 (0.41, 0.70)	0.60 (0.46, 0.73)	0.030	1.29	0.35	0.94
Hematological	0.61 (0.52, 0.70)	0.63 (0.54, 0.73)	0.090	1.00	0.42	0.83
Circulation	0.80 (0.75, 0.86)	0.82 (0.78, 0.87)	0.001	0.49	0.70	0.82
Neurologic	0.60 (0.53, 0.68)	0.65 (0.57, 0.72)	0.005	0.71	0.51	0.78
Digestive	0.75 (0.66, 0.85)	0.80 (0.72, 0.88)	0.014	0.71	0.76	0.74
Neoplasm	0.65 (0.53, 0.76)	0.66 (0.55, 0.77)	0.248	0.53	0.54	0.72
Respiratory	0.64 (0.58, 0.69)	0.66 (0.60, 0.72)	0.003	0.60	0.61	0.67
Trauma	0.77 (0.68, 0.86)	0.79 (0.71, 0.88)	0.023	1.85	0.64	0.93
Others	0.73 (0.65, 0.80)	0.81 (0.75, 0.86)	< 0.001	0.69	0.77	0.74

There was a better predictive value for the L/A ratio predicting hospital mortality than lactate in critically ill children with or without bacteremia (without bacteremia: 0.76, 95% CI 0.73–0.78 vs. 0.72, 95% CI 0.69–0.75, *P* < 0.001; with bacteremia: 0.63, 95% CI 0.58–0.67 vs. 0.60, 95% CI 0.55–0.65, *P* = 0.006). Our findings indicated that in the different age groups, the AUROC of the L/A ratio for predicting hospital mortality was higher than that of lactate, which suggested that the L/A ratio was applicable for predicting the prognosis of critical illness in children of different ages. Moreover, according to plasma levels of lactate, participants were divided into three groups, including the normal (< 2.0 mmol/L), intermediate (≥ 2.0 to < 4.0 mmol/L), and high lactate groups (≥ 4.0 mmol/L). Referring to Table 4, the L/A ratio had a higher AUROC for predicting hospital mortality than lactate, regardless of plasma lactate levels. The AUROC of the L/A ratio in different diseases was higher than that of lactate for predicting hospital mortality, but the differences in hematological diseases (*P* = 0.090) and neoplasms (*P* = 0.248) were not statistically significant.

**Fig. 2:**
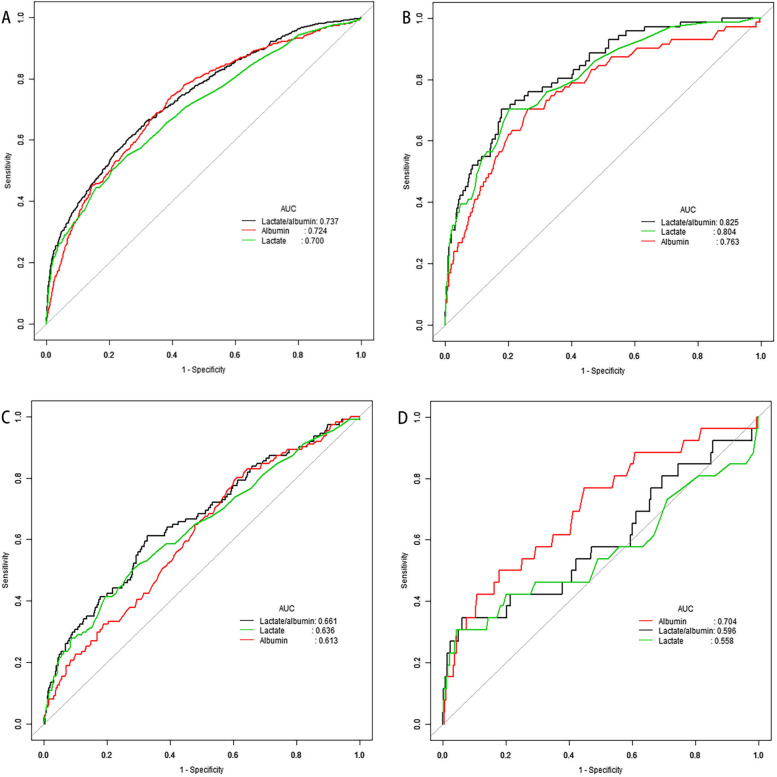
Receiver operating characteristic (ROC) curves of the lactate/albumin (L/A) ratio, lactate, and albumin for predicting in-hospital mortality. **A** All critically ill patients. **B** Patients with circulation disease. **C** Patients with respiratory disease. **D** Patients with congenital disease

Figure 2 shows the analysis results of the ROC curve of the L/A ratio, lactate, and albumin for predicting in-hospital mortality of the most common illnesses in our study (circulation, respiratory, and congenital diseases). In all critically ill patients, the AUC of the L/A ratio was higher than that of lactate and albumin (L/A ratio, 0.737; lactate, 0.700; and albumin, 0.724) (Fig. 2A), indicating that the L/A ratio had better prognostic ability in critical illness than lactate and albumin alone. Similarly, in the critical circulatory and respiratory diseases, the L/A ratio showed superiority in predicting the prognosis of critical illness than lactate and albumin (circulatory disease: L/A ratio 0.825, lactate 0.804, albumin 0.763; respiratory disease: L/A ratio 0.661, lactate 0.636, and albumin 0.613) (Figs. 2B and C). However, in congenital diseases, albumin had a higher AUC than the L/A ratio (albumin 0.704, L/A ratio 0.596) (Fig. 2D).

### Subgroup analysis of the association between the L/A ratio and in-hospital mortality

**Fig. 3:**
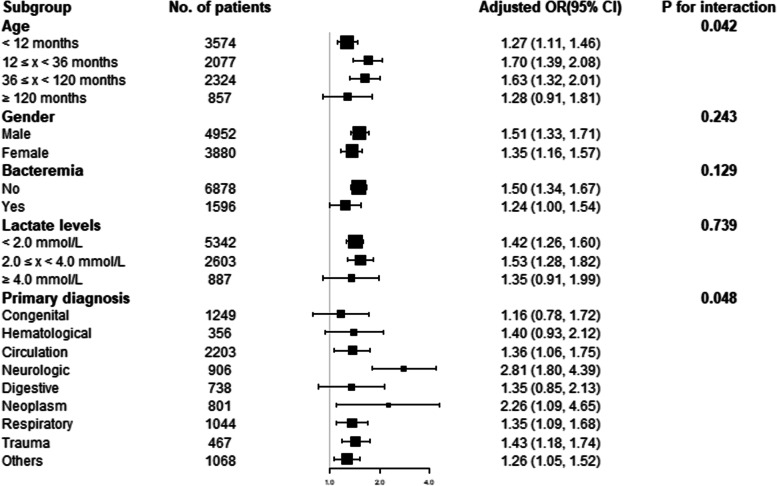
Subgroup analysis of the association between in-hospital mortality and the lactate/albumin (L/A) ratio

We used age, sex, bacteremia, lactate levels, and primary diagnosis as stratification variables to analyze the relationship between the L/A ratio and in-hospital mortality (Table 5). There was no association between L/A ratio and hospital mortality in the age ≥ 120 months, lactate levels ≥ 4.0 mmol/L, congenital diseases, hematological diseases and digestive diseases subgroups (Fig. 3). L/A ratio was most strongly associated with hospital mortality in the age 12–36 months group (OR 1.70, 95% CI 1.39–2.08). Elevated lactate levels had the strongest association with hospital mortality in the 36–120 months group (OR 1.28, 95% CI 1.18–1.37). In different lactate levels subgroups, strong associations between the L/A ratio, lactate, and hospital mortality were found in the intermediate lactate levels (2.0–4.0 mmol/L) (L/A ratio: OR 1.53, 95% CI 1.28–1.82; lactate: OR 1.17, 95% CI 1.10–1.25). There was a strong association between the L/A ratio, lactate, and hospital mortality in neurological diseases (L/A ratio: OR 2.81, 95% CI 1.80–4.39; lactate: OR 1.40, 95% CI 1.22–1.62).

**Table 5:** Subgroup analysis of the association between lactate/albumin ratio, lactate and in-hospital mortality

	N	Lactate/albumin ratio	*P* for interaction	Lactate	*P* for interaction
Age			0.042		< 0.001
< 12 months	3574	1.27 (1.11, 1.46)		1.09 (1.04, 1.15)	
12 ≤ x < 36 months	2077	1.70 (1.39, 2.08)		1.23 (1.15, 1.32)	
36 ≤ x < 120 months	2324	1.63 (1.32, 2.01)		1.28 (1.18, 1.37)	
≥ 120 months	857	1.28 (0.91, 1.81)		1.13 (1.01, 1.27)	
Gender			0.243		0.061
Male	4952	1.51 (1.33, 1.71)		1.19 (1.14, 1.25)	
Female	3880	1.35 (1.16, 1.57)		1.12 (1.06, 1.18)	
Bacteremia			0.129		0.211
No	6878	1.50 (1.34, 1.67)		1.18 (1.13, 1.22)	
Yes	1596	1.24 (1.00, 1.54)		1.11 (1.03, 1.20)	
Lactate levels			0.739		0.705
Normal lactate (< 2.0 mmol/L)	5342	1.42 (1.26, 1.60)		1.17 (1.12, 1.22)	
Intermediate lactate (2.0 ≤ x < 4.0 mmol/L)	2603	1.53 (1.28, 1.82)		1.17 (1.10, 1.25)	
High lactate ( ≥ 4.0 mmol/L)	887	1.35 (0.91, 1.99)		1.10 (0.96, 1.27)	
Primary diagnosis			0.048		0.040
Congenital	1249	1.16 (0.78, 1.72)		1.05 (0.92, 1.18)	
Hematological	356	1.40 (0.93, 2.12)		1.10 (0.94, 1.27)	
Circulation	2203	1.36 (1.06, 1.75)		1.12 (1.04, 1.22)	
Neurologic	906	2.81 (1.80, 4.39)		1.40 (1.22, 1.62)	
Digestive	738	1.35 (0.85, 2.13)		1.13 (0.97, 1.33)	
Neoplasm	801	2.26 (1.09, 4.65)		1.28 (1.05, 1.56)	
Respiratory	1044	1.35 (1.09, 1.68)		1.13 (1.05, 1.21)	
Trauma	467	1.43 (1.18, 1.74)		1.25 (1.14, 1.36)	
Others	1068	1.26 (1.05, 1.52)		1.14 (1.04, 1.24)	

Adjusted for age, sex, ICU type, bacteremia, vasopressors use, WBC, PLT, hemoglobin, ALT, CK-MB, sodium, INR and CRP

## Discussion

Early evaluation of the prognosis of critical illness is conducive to the accurate stratification of different critical illness according to the severity of the disease to take different treatment measures. The present study indicated that the L/A ratio is of great value as a prognostic marker of pediatric ICU in-hospital mortality in critically ill patients aged 28 days to 18 years. Higher L/A ratio, higher lactate, and lower albumin levels were found in the non-survivor group than in the survivor group in critically ill children. Several studies showed that these trends were associated with poor prognosis in adult critical care patients [2, 22, 23]. This indicates that lactate, albumin, and the L/A ratio may also play important roles in predicting prognosis in critically ill children.

Logistic regression indicated that L/A ratio and lactate levels were independent risk factors and albumin was a protective factor for critical illness in children, with L/A ratio showing the strongest association with in-hospital mortality. ROC analysis was performed to evaluate the prognostic value of the L/A ratio and lactate levels in children with critical illnesses, which indicated that the overall AUROC of the L/A ratio for predicting in-hospital mortality was higher than that of lactate alone. The results of logistic regression and ROC analysis both can indicate that the L/A ratio has a great value in predicting the prognosis of critical illness in children, superior to lactate and albumin. The overall cut-off value for the L/A ratio in our study was 0.55, demonstrating that patients with a L/A ratio > 0.55 had a worse prognosis. This cut-off value was slightly lower than that in another study [22], which reported that the cut-off value of critically ill patients (age ≥ 15) was 1.01. We believe the age was responsible for this difference. Firstly, adult patients in ICU usually have more complex complications than pediatric patients, such as hypertension, diabetes and connective tissue disease. Secondly, the courses of illness in adult patients are generally longer than that of pediatric patients. The above-mentioned factors result in lactate accumulation and albumin consumption, and therefore a higher L/A ratio in adult patients compared to children is observed.

Logistic regression analysis indicated that there was an evident association between L/A ratio and hospital mortality of patients aged < 120 months. The AUROC of the L/A ratio for predicting hospital mortality was higher than that of lactate in the patients aged < 120 months. We suggested that when the patients were younger than 120 months, the L/A ratio was better than lactate to predict the prognosis of critical illness in children. Logistic regression analysis showed that the L/A ratio and lactate were associated with in-hospital mortality of patients with bacteremia. The ROC analysis showed a better predictive value for the L/A ratio in predicting hospital mortality in patients with bacteremia than lactate levels alone, similar to the results in adults with sepsis [23]. Some studies have reported that hyperlactatemia (> 2 mmol/L) is an independent predictor of mortality in critically ill patients [24, 25]. To detect whether the prognostic value of higher plasma lactate levels was better than that of the L/A ratio, we divided participants into three groups: normal, intermediate, and high lactate groups. Logistic regression showed that the increased L/A ratio was associated with increased hospital mortality in lactate levels < 4.0mmol/L. Regardless of plasma lactate levels, there was a higher AUROC in the L/A ratio than in lactate for predicting hospital mortality. Similarly, Chebl et al. found that the AUC of the L/A ratio was significantly higher than that of lactate alone, regardless of lactate level (< 2 mmol/L or > 2 mmol/L) [23]. Thus, we suggested that the L/A ratio can be an independent risk factor and a better prognostic biomarker than lactate alone in children with critical illnesses with lactate levels < 4.0mmol/L. Furthermore, we analyzed the predictive value of L/A ratio and lactate in different primary diseases. Logistic regression showed that there were no associations between the L/A ratio, lactate levels, and hospital mortality in some primary diseases, such as congenital, hematological, and digestive diseases. ROC analysis showed that the L/A ratio had a better predictive value than lactate levels for many critical illnesses except neoplasm and hematological diseases. Wang et al. reported that the L/A ratio could predict multiple organ dysfunction syndromes (MODS) [2]. We suggested that L/A ratio has a better value in predicting the prognosis of critical illness in children than lactate in circulatory, respiratory and trauma diseases.

Our study demonstrated that lactate levels were higher in non-survivors than in survivors. The AUC of lactate in predicting mortality of patients with critical illness was 0.70, which supported that lactate can also act as an effective prognostic biomarker in the prognosis of critically ill children. Multivariable logistic regression indicated that the lactate level was an independent predictor of prognosis in critically ill children. Previous studies have reported that plasma lactate levels are related to a poor prognosis of severe illness [26–29]. But lactate levels also elevate in the non-pathological conditions as mentioned before. We found that albumin levels were lower in non-survivors than in survivors. Logistic regression showed that the albumin level acts as a protective factor of critical illness in children. Albumin is often used to predict the prognosis of severe illness and health outcomes in chronic and inflammatory diseases [30, 31] and is associated with vulnerability to stressors, unstable homeostasis, and debility. The degree of hypoalbuminemia correlates with the intensity of the inflammatory response in critically ill patients [32]. However, albumin levels are affected by multiple factors such as nutritional status and chronic inflammation.

Above all, it is not reasonable to evaluate and predict the prognosis of critically ill patients using lactate or albumin levels alone. The L/A ratio has a great predictive value, which takes both inflammation conditions and nutritional status into consideration. Biomarkers and associated clinical information can easily be collected when patients are admitted to the PICU, making the application of the L/A ratio in critical illness possible, which is helpful for diagnosis, treatment, and prediction of the prognosis of critical illness in children.

## Limitation

Although our study included a large sample size, which can minimize sampling error, it is a single-center retrospective study that is not verified in real clinical conditions, restricting its generalizability to other medical environments. Furthermore, lactate and albumin levels are affected by multiple factors, therefore, patients’ overall situation must be considered when using the L/A ratio to predict the prognosis of critical illness in children.

## Conclusions

In summary, the L/A ratio is a useful predictor with great prognostic performance for in-hospital mortality in critically ill children. In the future, more prospective studies should be conducted to test the predictive value of the L/A ratio in critical illness.

## Supplementary Information

**Additional file 1:** **Supplementary Table 1.** Relationship between the variables and in-hospital mortality in different models of multivariable logistic regression.

## Data Availability

The datasets generated and/or analysed during the current study are available in the pediatric intensive care (PIC) database (version 1.1.0), http://pic.nbscn.org/

## Abbreviations

PICU: pediatric intensive care units
L/A: lactate/albumin
PIC: pediatric intensive care
AUROC: area under the receiver operating characteristic curve
OR: odds ratio
CI: confidence interval
