# Supplementary material for: Lactate/albumin ratio as a predictor of in-hospital mortality in critically ill children
Source: BMC Pediatr. 2022 Dec 20;22:725. doi: 10.1186/s12887-022-03787-0 (PMC9764537; doi:10.1186/s12887-022-03787-0)
Supplement: Supplementary file 1 — Additional file 1: Supplementary Table 1. Relationship between the variables and in-hospital mortality in different models of multivariable logistic regression. [file 12887_2022_3787_MOESM1_ESM.docx]

**Supplementary Table 1** Relationship between the variables and in-hospital mortality in different models of multivariable logistic regression.

|  | Model I | | | Model II | | |
| --- | --- | --- | --- | --- | --- | --- |
|  | OR | 95% CI | *P* | OR | 95% CI | *P* |
| Age | 1.00 | (1.00, 1.00) | 0.263 | 1.00 | (0.99, 1.00) | 0.001 |
| Gender |  | | | | | |
| Female | Reference | | | Reference | | |
| Male | 1.32 | (1.09, 1.60) | 0.004 | 1.27 | (1.00, 1.60) | 0.048 |
| Bacteremia |  | | | | | |
| No | Reference | | | Reference | | |
| Yes | 2.18 | (1.78, 2.67) | <0.001 | 1.24 | (0.96, 1.59) | 0.096 |
| Vasopressors use |  | | | | | |
| No | Reference | | | Reference | | |
| Yes | 5.12 | (3.44, 7.60) | <0.001 | 7.67 | (4.71, 12.47) | <0.001 |
| Unknown | 3.21 | (2.15, 4.78) | <0.001 | 12.74 | (7.10, 22.86) | <0.001 |
| ICU type |  | | | | | |
| CICU | Reference | | | Reference | | |
| GICU | 12.03 | (7.91, 18.29) | <0.001 | 55.01 | (31.21, 96.95) | <0.001 |
| NICU | 4.69 | (2.58, 8.53) | <0.001 | 5.41 | (2.67, 10.97) | <0.001 |
| PICU | 9.41 | (6.15, 14.38) | <0.001 | 7.43 | (4.51, 12.23) | <0.001 |
| SICU | 1.81 | (1.11, 2.95) | 0.018 | 2.01 | (1.16, 3.45) | 0.012 |
| L/A ratio | 2.02 | (1.86, 2.19) | <0.001 | 1.44 | (1.31, 1.59) | <0.001 |
| Lactate | 1.27 | (1.23, 1.30) | <0.001 | 1.16 | (1.12, 1.21) | <0.001 |
| Albumin | 0.90 | (0.89, 0.91) | <0.001 | 0.95 | (0.93, 0.97) | <0.001 |
| WBC | 1.00 | (1.00, 1.01) | <0.001 | 1.00 | (1.00, 1.00) | 0.941 |
| PLT | 1.00 | (1.00, 1.00) | <0.001 | 1.00 | (1.00, 1.00) | 0.008 |
| Hemoglobin | 0.98 | (0.98, 0.99) | <0.001 | 1.00 | (1.00, 1.01) | 0.139 |
| ALT | 1.00 | (1.00, 1.00) | <0.001 | 1.00 | (1.00, 1.00) | 0.595 |
| CK-MB | 1.00 | (1.00, 1.00) | <0.001 | 1.00 | (1.00, 1.00) | 0.112 |
| Sodium | 1.02 | (1.00, 1.04) | 0.039 | 1.01 | (0.99, 1.02) | 0.524 |
| INR | 1.96 | (1.75, 2.19) | <0.001 | 1.19 | (1.04, 1.35) | 0.012 |
| CRP | 1.01 | (1.00, 1.01) | <0.001 | 1.00 | (1.00, 1.01) | 0.002 |

OR, odds ratio; CI, confidence interval; ICU, intensive care unit; CICU, cardiac intensive care unit; GICU, general intensive care unit; NICU, neonatal intensive care unit; PICU, pediatric intensive care unit; SICU, surgical intensive care unit; L/A ratio, **lactate/albumin ratio;** WBC, white blood cell; PLT, platelet; ALT, alanine transaminase; CK-MB, c**reatine kinase-MB;** INR, **international normalized ratio;** CRP, C reactive protein.
